# Design of Novel IRAK4 Inhibitors Using Molecular Docking, Dynamics Simulation and 3D-QSAR Studies

**DOI:** 10.3390/molecules27196307

**Published:** 2022-09-24

**Authors:** Swapnil P. Bhujbal, Weijie He, Jung-Mi Hah

**Affiliations:** 1 Department of Pharmacy, College of Pharmacy, Hanyang University, Ansan 426-791, Korea; 2 Institute of Pharmaceutical Science and Technology, Hanyang University, Ansan 426-791, Korea; 3 Second Affiliated Hospital of Shantou University Medical College, Shantou University, Shantou 515000, China

**Keywords:** autoimmune disease, cancer, IRAK4, inhibitor, MM-PBSA, molecular docking, dynamics simulation, 3D-QSAR

## Abstract

Treatment of several autoimmune diseases and types of cancer has been an intense area of research over the past two decades. Many signaling pathways that regulate innate and/or adaptive immunity, as well as those that induce overexpression or mutation of protein kinases, have been targeted for drug discovery. One of the serine/threonine kinases, Interleukin-1 Receptor Associated Kinase 4 (IRAK4) regulates signaling through various Toll-like receptors (TLRs) and interleukin-1 receptor (IL1R). It controls diverse cellular processes including inflammation, apoptosis, and cellular differentiation. MyD88 gain-of-function mutations or overexpression of IRAK4 has been implicated in various types of malignancies such as Waldenström macroglobulinemia, B cell lymphoma, colorectal cancer, pancreatic ductal adenocarcinoma, breast cancer, etc. Moreover, over activation of IRAK4 is also associated with several autoimmune diseases. The significant role of IRAK4 makes it an interesting target for the discovery and development of potent small molecule inhibitors. A few potent IRAK4 inhibitors such as PF-06650833, RA9 and BAY1834845 have recently entered phase I/II clinical trial studies. Nevertheless, there is still a need of selective inhibitors for the treatment of cancer and various autoimmune diseases. A great need for the same intrigued us to perform molecular modeling studies on 4,6-diaminonicotinamide derivatives as IRAK4 inhibitors. We performed molecular docking and dynamics simulation of 50 ns for one of the most active compounds of the dataset. We also carried out MM-PBSA binding free energy calculation to identify the active site residues, interactions of which are contributing to the total binding energy. The final 50 ns conformation of the most active compound was selected to perform dataset alignment in a 3D-QSAR study. Generated RF-CoMFA (*q*^2^ = 0.751, ONC = 4, *r*^2^ = 0.911) model revealed reasonable statistical results. Overall results of molecular dynamics simulation, MM-PBSA binding free energy calculation and RF-CoMFA model revealed important active site residues of IRAK4 and necessary structural properties of ligand to design more potent IRAK4 inhibitors. We designed few IRAK4 inhibitors based on these results, which possessed higher activity (predicted pIC_50_) than the most active compounds of the dataset selected for this study. Moreover, ADMET properties of these inhibitors revealed promising results and need to be validated using experimental studies.

## 1. Introduction

One of the significant serine/threonine kinases, IRAK4 (Interleukin-1 receptor associated kinase 4), is essential for the scaffolding and phosphorylation of the toll-like receptor (TLR) [1,2]. IRAK1, IRAK2, IRAK-M, and IRAK4 are all homologs of the IRAK protein and they have an N-terminal death domain, a linker, and a kinase domain; however, only IRAK4 lacks a C-terminal extension [3,4]. IRAK4 kinase domain contains typical two lobe conformation observed in several protein kinases with the ATP binding pocket. The N-terminal lobe of IRAK4 has two distinctive features: An N-terminal extension of unknown function and a tyrosine as the gatekeeper residue [3,4]. Usually, in the ATP-binding pocket, the key gatekeeper is responsible for providing access to the pre-existing hydrophobic pocket shaped by the residues of α helix C, DFG motif, and the gatekeeper residue. The existence of a tyrosine gatekeeper, which is unique to the IRAK family, has significance for the development of selective IRAK4 inhibitors [3,5].

IRAKs are important mediators of interleukin-1 receptor (IL1R) and TLR signalling processes including IL-33, IL-18, and IL-1 receptors [2]. TLR/IL1R-mediated signalling regulates multiple cellular processes such as apoptosis, inflammation, and cellular differentiation. TLR/IL1R signalling is associated with the employment of adaptor molecules such as Mal/TIRAP, MyD88, TRAM and TRIF that play crucial role in the operating and activation of IRAK family [3,6]. When IRAK4 is recruited to MyD88, activation of IRAK4 causes IRAK1 and/or IRAK2 to be phosphorylated [7]. The MyD88:IRAK4:IRAK2 het-eromeric structure is known the myddosome complex. When the myddosome complex is formed, IRAK1 phosphorylation initiates the accomplishment of scaffolding with TRAF6 that further induces downstream signaling such as NFκB-mediated transcription activation [8].

In combination with B cell and T cell hyperactivity, abnormal IRAK4 activation stimulates the inflammatory chemokine and cytokine pathways, which increases autoimmune signaling in the associated disease [9]. Other study disclosed that human patients missing IRAK4 are seriously hyporesponsive and immunocompromised to IL-1 and LPS [10]. Therefore, IRAK4 has a vital role in innate immunity and its inhibition by small molecules would be significant for the treatment of various types of inflammations and autoimmune disease such as inflammatory bowel disease (IBD), rheumatoid arthritis (RA) and Systematic Lupus Erythematosus (SLE) [8,11].

Moreover, IRAK4 has a vital role in the growth of several malignancies [11]. Patients with melanoma have been found to have higher basal levels of IRAK4 phosphorylation [12]. MyD88 gain-of-function mutations (L265P somatic mutation) lead to numerous rare and difficult to treat hematological malignancies such as activated B cell diffuse large B-cell lymphoma (ABC-DLBCL), Waldenström macroglobulinemia (WM), chronic lymphocytic leukemia (CLL) and pancreatic ductal adenocarcinoma (PDAC) [13,14]. Aberrant activation of the NF-κB is also responsible for various cancer types, including colorectal cancer [15]. The evident role of IRAK4 inhibitors as probable anticancer and anti-inflammatory agents impelled us to perform several molecular modelling studies including 3D-QSAR toward the discovery of novel IRAK4 inhibitors. Although there have been previous reports aimed at the generation of IRAK4 inhibitors, the development of cancer drugs is currently of renewed interest.

Over the past few years, there have been dozens of patent applications as well as the peer reviewed literature filed/published appealing small molecule inhibitors of IRAK4 [16,17]. It seems that autoimmune and cancer diseases are being targeted. Few pharma companies have proposed their inhibitors, among which Pfizer is the most advanced, having completed a phase II clinical study with their candidate compound, PF-06650833 (IC_50_ = 0.2 nM) for rheumatoid arthritis (RA) and BAY 1834845 has entered phase 1 clinical trials [17,18]. Additionally, Aurigene/Curis and BMS companies proposed their compounds, CA-4948 (IC_50_ < 50 nM) and BMS-986126 (IC_50_ = 5.3 nM) respectively [16,17]. Each candidate small molecule has demonstrated reasonable efficacy in several murine autoimmune disease models and are currently in Phase I or II clinical trials [1,4]. However, the design of potent and selective IRAK4 inhibitors for the treatment of cancer is still required.

Hence, in the current study, we have implemented various molecular modelling studies on the diaminonicotinamide derivatives as IRAK4 anticancer inhibitors. Initially we executed molecular docking and 50 ns dynamics simulation (MD) of the most active compound **33** from selected dataset for this study [2], following MM-PBSA binding free energy calculations. These studies helped us to understand the binding mode of IRAK4 inhibitor and key active site residues of IRAK4, which contribute most to the total binding energy. We then performed (3D-QSAR) 3D-quantitative structure-activity relationship by using the 50 ns pose from MD to align dataset compounds into the binding site of receptor. Altogether, analyses of results revealed crucial structural characteristics of ligand to improve the potency. We designed few IRAK4 inhibitors that possess better-predicted activity (pIC_50_) than the most active compound of the dataset used in this study. Our design scheme and predicted ADMET values could be useful for medicinal chemists or pharmaceutical companies to develop novel IRAK4 inhibitors. Further experimental studies need to be performed to validate our designed IRAK4 inhibitors.

## 2. Results and Discussion

### 2.1. Molecular Docking

**Figure 1:**
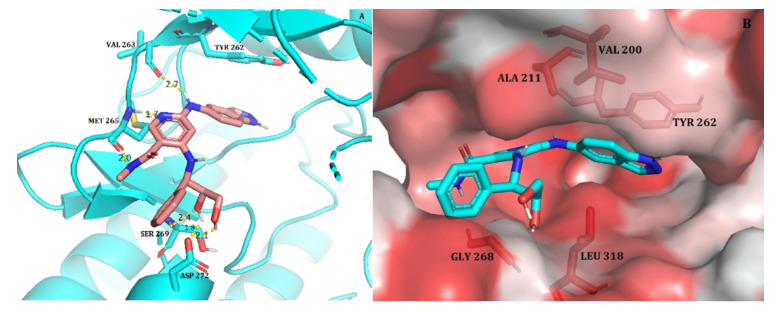
(**A**) Docked pose of the most active compound, 33, within the active site of IRAK4 (Hydrogen bonds are represented as yellow dotted lines); (**B**) The most active compound 33 (shown in stick model) within the hydrophobic pocket of IRAK4; the red coloured region represents the most hydrophobic surface of the protein, and the white colour represents the least hydrophobic surface. Hydrophobic residues are indicated with red sticks.

The co-crystalized ligand was re-docked into the IRAK4 active site to evaluate the docking technique. The co-crystallized ligand and the re-docked ligand had similar binding conformations and H-bond interactions. Their difference in RMSD (root mean square deviation) was 1.20 Å that proved the docking technique was reliable. Then we docked the most active compound 33 from selected dataset into the active site of IRAK4 (Figure 1A). Based on the lowest binding energy and binding interactions with the active site residues, compound 33′s docked pose was chosen. Binding energy of the compound 33 with IRAK4 was found to be -7.94 kcal/mol which showed six hydrogen bonds. Three hydrogen bonds were formed with the key hinge region residues. The NH from methylnicotinamide and pyridyl nitrogen formed two hydrogen bonds with residue Met265. Third hinge region hydrogen bond was observed between residue Val263 and the NH of amide. These three hydrogen bonds are known as ‘classical triad hinge binding interaction’, which are important interactions and were seen in docking of co-crystallized ligand as well as other IRAK4 inhibitors [2]. The two OH groups from phenylbutane-1,2-diol formed 3 hydrogen bonds with residues Ser269 and Asp272 respectively. Furthermore, indoline ring was docked into the hydrophobic pocket and it formed pi-pi interaction with the gatekeeper residue Tyr262. The interaction with Tyr262 was reported to be unique and essential for the inhibition of IRAK4 [2].

The docked pose of compound 33 was further analyzed to assess hydrophobic interactions. A Python script ‘colour h’ was utilized to colour the hydrophobic residues of IRAK4 and check their interactions with compound 33. This script uses an Eisenberg hydrophobicity scale (Figure 1B) to colour the receptor in PyMOL [19]. The most hydrophobic residues were colored red, and the least hydrophobic area was colored white. The amide group and indoline ring are docked deep inside the hydrophobic pocket occupying residues Val200, Ala211, Leu318 and Tyr262. As discussed earlier, Tyr262 is one of the im-portant residue in the active site of IRAK4 and unique to the IRAK family as a gatekeeper residue, interaction of which makes it crucial for IRAK4 inhibition. It was also reported that the interaction with Tyr262 plays an efficient role in selectivity over other kinases such as JAK3 [2]. Therefore, based on observed important interactions of compound 33 with IRAK4 and its low binding energy, this pose was selected for further validation using molecular dynamics simulation.

### 2.2. Molecular Dynamics Simulation

**Figure 2:**
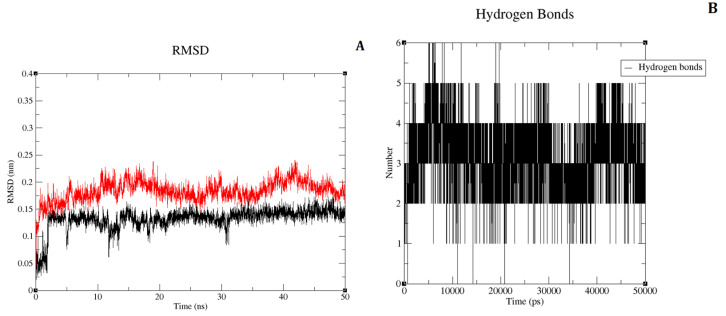
(**A**) Root mean square deviations (RMSDs) of the protein and compound 33, lig-and RMSD is shown in black color and protein RMSD in red color; (**B**) The graph of hydrogen bonds between compound 33 and IRAK4 throughout the 50 ns of MD.

To examine the binding stability and conformation of the ligand, Gromacs-2018 [20] was used to conduct MD simulation of the docked complex of compound 33 and IRAK4. A production run of 50 ns MD simulation was performed. Figure 2A shows the root mean square deviation (RMSD) for a ligand and protein. The protein RMSD and ligand RMSD are shown in red and black colored lines in the graph, respectively. The plot indicates that the protein’s RMSD approached stability at 20 ns, varied somewhat between 25 and 40 ns, and then stabilized at the end of the simulation, indicating that the protein’s stable conformation was attained. Prior to the 50 ns simulation, no variations with less than 0.1 nm deviations were seen in compound 33′s RMSD, which stabilised at 18 ns. During the simulation, there were hardly any fluctuations seen, with the exception of the loop sections, and the inclusive fluctuation was less than 2Å. At the end of the simulation, the system was in equilibrium, according to overall RMSD analyses.

**Figure 3:**
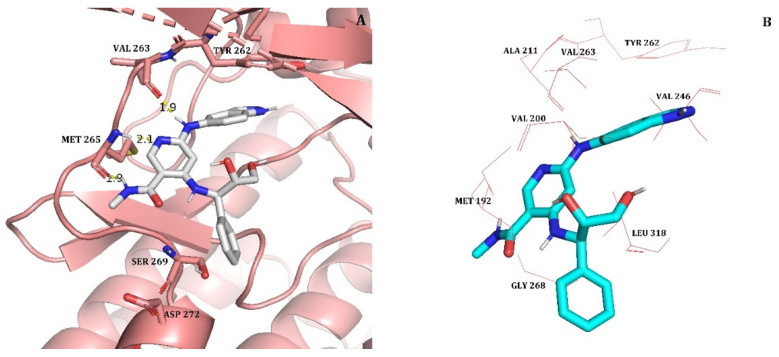
(**A**) 50 ns MD conformation of the most active compound 33 (shown in white color and stick model) inside the active site of IRAK4. Hydrogen bonds are represented as yellow dotted lines and their distances are labeled in angstrom. (**B**) The most active compound 33 (shown in cyan, stick model) inside the hydrophobic pocket of IRAK4; surrounded by hydrophobic residues (red colored line representation).

Hydrogen bond analysis of compound 33 at 50 ns showed that it formed 3 H-bonds with the crucial residues of IRAK4 from hinge region (Figure 3A). These 3 hydrogen bonds with residues Val263 and Met265 (classical triad hinge binding interaction) are same as that of hydrogen bonds observed in the docking analysis above. These three interactions were consistent throughout the 50 ns of simulation (Figure 2B). Therefore, 50 ns pose of compound 33 retained three crucial hydrogen bonds with IRAK4. The pi-pi interaction of indoline with Tyr262 was also observed. But other 3 hydrogen bonds with residues Ser269 and Asp272 were lost after 50 ns MD simulation due to the change in the conformation of phenylbutane-1,2-diol moiety. This conformational change was expected due to a special tyrosine gatekeeper residue, IRAK4 lacks a rear pocket in the ATP binding site, and its solvent-exposed region is larger than that of other kinases [2]. It was also reported previously that efforts to strengthen the H-bond to the carboxylic acid of Asp272 were less successful [2]. However, pi-pi interaction and important hydrophobic interactions with residues Tyr262, Val200, Ala211 and Leu318 were reproduced at the end of 50 ns MD simulation (Figure 3B). Hence, we considered 50 ns pose as a reasonable binding pose of compound 33 and used it as a template structure for the alignment of dataset compounds in 3D-QSAR studies.

### 2.3. MM/PBSA Binding Free Energy Calculation

**Figure 4:**
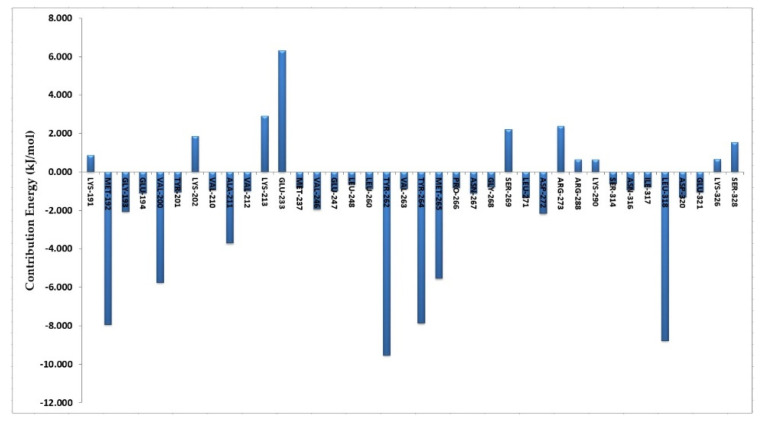
The column chart depicting the each residue contribution in the total binding free energy of compound 33.

The MM/PBSA package [21] was utilized to compute the binding affinity of compound 33. The predicted binding free energy was -112.841 kJ/mol. It is the sum of Van der Waal energy of -220.417 kJ/mol, electrostatic energy of -45.771 kJ/mol, polar salvation energy of 173.117 kJ/mol and SASA energy of -19.859 kJ/mol. Van der Waals energy and electrostatic energy were important for compound 33′s binding with IRAK4. However, the binding of component 33 did not benefit from the polar salvation energy. In our docking and MD analyses, most of the interactions formed by compound 33 were hydrogen bonds and hydrophobic that were found to be consistent. This explains why Van der Waals energy contributed the most among them. Additionally, for a thorough understanding of the ligand-protein interactions, we carried out a binding free energy decomposition analysis. The energy decomposition of each residue is depicted in column chart (Figure 4). The main contribution to the binding of compound 33 was from residues Tyr262, Leu318, Tyr264, Met265, Ala211 and Val263, which were involved in the hydrogen bond and hydrophobic interactions. On the contrary, residues Glu238 and Lys213 were in disfavor with the binding of compound 33. In conclusion, the study of the binding free energy demonstrated the role of essential active site residues in IRAK4 inhibition.

### 2.4. 3D-QSAR (CoMFA and RF-CoMFA)

**Figure 5:**
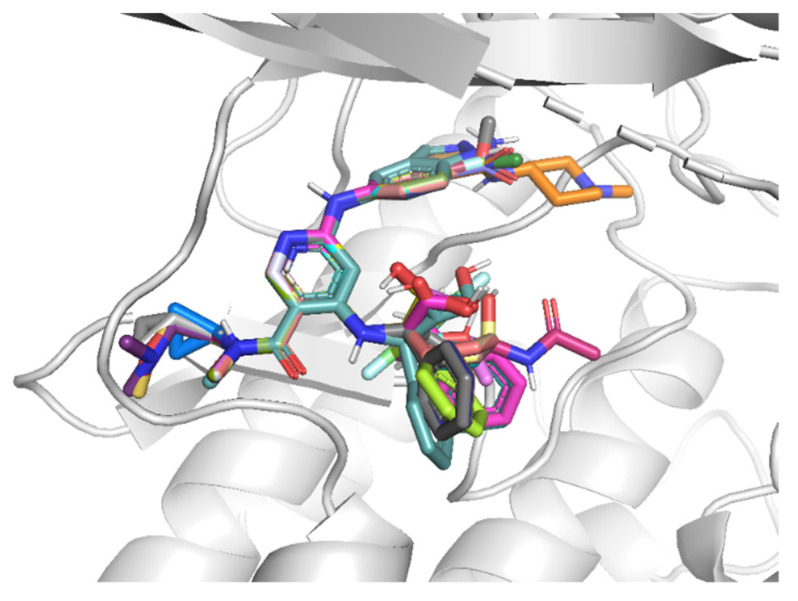
Alignment of the dataset compounds inside the active site of IRAK4.

Receptor-based comparative molecular field analysis (CoMFA) and region focused CoMFA (RF-CoMFA) [22] models were developed for the diaminonicotinamide derivatives. All dataset compounds were sketched and aligned inside the active site of IRAK4 using the MD conformation of the most active compound 33 as a template in SYBYL-X 2.1. The alignment of the dataset compounds is shown in Figure 5. The dataset compounds were separated into training set (26) and test set (12) using the standards given by Golbraikh et al. and algorithm 4 (activity ranking) in the reported article [23]. We chose algorithm 4 (activity ranking) because there are no large gaps in activity values of dataset compounds and algorithm 4 can construct a test set that represents the whole range of activities. Thus, our test set contains compounds having high, medium, and low activity (pIC_50_) values.

For assessing the reliability of a 3D-QSAR model, it is essential to calculate several statistical parameters using the partial least square (PLS) method, such as cross-validated correlation coefficient (*q*^2^), non-cross-validated correlation coefficient (*r*^2^), standard error of estimate (SEE), optimal number of components (ONC), and F value. We developed CoMFA models (*q*^2^ = 0.502, ONC = 4, *r*^2^ = 0.823) for the full dataset. These statistical values were in an acceptable range but quite low to consider them as a good predictive model. Therefore, we derived region focused CoMFA by using the PLS analysis obtained in the CoMFA model (RF-CoMFA: *q*^2^ = 0.527, ONC = 6, *r*^2^ = 0.905). The obtained RF-CoMFA model possessed better statistical results hence it was selected for further validation. RF-CoMFA model derived using external test set validation (*q*^2^ = 0.751, ONC = 4, *r*^2^ = 0.911) showed highest *q*^2^ and *r*^2^ values. The latter model was selected as a final model due to its better *q*^2^ and *r*^2^ values. The detailed statistical values of the selected RF-CoMFA model are given in Table 1.

#### Validation of RF-CoMFA Model

**Figure 6:**
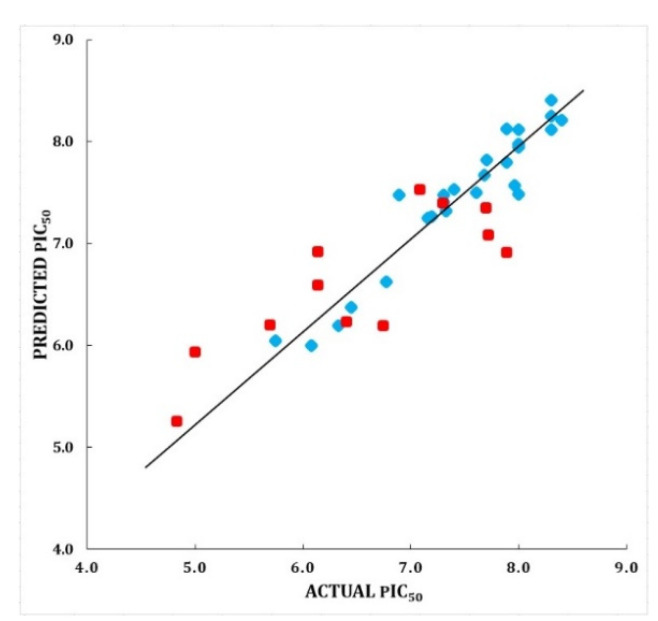
Scatter plot for the selected RF-CoMFA model; the plot shows the actual pIC_50_ versus predicted pIC_50_ activity of the training and test sets; the training set compounds are represented as blue diamonds; the test set compounds are represented as red squares.

A number of validation techniques were used to assess robustness and predictive ability of produced CoMFA model. All the techniques, such as bootstrapping, predictive *r*^2^ (external test set), progressive scrambling (*Q*^2^), and *rm*^2^ metric calculation, exhibited statistical values that were within the adequate range [24,25]. These findings showed that the chosen model was reliable and predictive. Detailed statistical values are shown in Table 1. In Table S2 of the Supplementary Material, the experimental and predicted activity values for this model are presented. The scatter plot for the same is shown in Figure 6. The compound 33 is shown superimposed with RF-CoMFA contour maps into the active site of IRAK4.

### 2.5. Contour Map Analysis

#### RF-CoMFA Contour Maps

**Figure 7:**
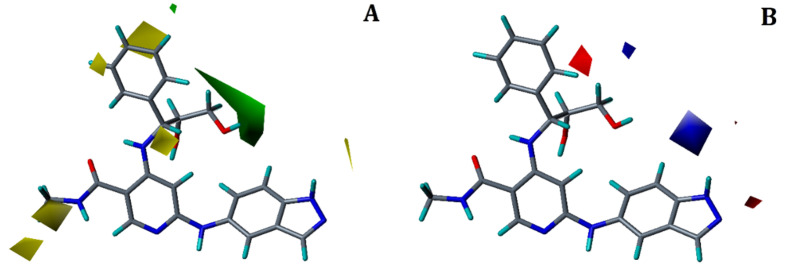
Contour maps for the selected RF-CoMFA model. (**A**) Steric contour map; (**B**) electrostatic contour map green contour shows the regions favorable for bulky substitutions and yellow contours shows the regions unfavorable for bulky substitutions; Blue contour favors electropositive substitutions while red contour favors electronegative substitutions.

Figure 7 displays the steric and electrostatic contour maps of the RF-CoMFA model. Green and blue colors indicate locations that are suitable for steric and electropositive substitutions, while yellow and red colors indicate regions that are unfavorable for these types of replacements.

In steric contour map, a big green-colored contour (Figure 7A) was observed at R^2^ position of the propan-1,2-diol moiety, indicating that in this area, bulky groups are preferred to elevate the activity. Substituting a steric group at R^2^ position could interact with many hydrophobic pocket residues of IRAK4. The hydrophobic interactions with residues Leu318 and Gly268 seen in our docking and MD simulation study of compound 33 can help to explain this. Similarly, two small yellow colored contours were present near both phenyl ring of the R^2^ substitution and methylacetamide at R^1^ position, which suggests that this region is unfavorable for the bulky groups. Adding bulky group at these positions may hinder with the hinge region residues. In the electrostatic contour map (Figure 7B), a small blue colored contour was located near the NH of indoline ring at the R^3^ position, which shows that the electropositive group in this position is favorable and may interact via H-bonds with neighboring active site residues. Conversely, a small red colored contour was seen near phenyl ring at R^2^ substitution; signifying that electronegative groups at this place are favorable. Thus, overall contour map analysis, docking and MD simulation analyses revealed important structural features of a ligand to improve their potency.

### 2.6. Designing of IRAK4 Inhibitors and Their ADMET Calculation

A reliable RF-CoMFA model development and its contour map analysis was valuable to propose a design strategy to design more potent IRAK4 compounds (Table 2). The structural characteristics studied and analyzed from contour maps were used to design new IRAK4 inhibitors. Using this strategy, we designed a small number of inhibitors, whose activities were then predicted using the chosen RF-CoMFA model. All designed compounds exhibited predicted activity (pIC_50_) more than the activity of the most potent compound 33 of the dataset. In Table 2, the proposed compounds’ structures and predicted pIC_50_ values are displayed. 

Additionally, we predicted the ADMET characteristics for each designed compound. Table 3 displays their properties in detail. We have predicted in silico ADMET (absorption, distribution, metabolism, excretion and toxicity), physicochemical properties, pharmacokinetics, drug-likeness and medicinal chemistry friendliness using Artificial Intelligence based In-silico ADME/Tox prediction online tool (https://www.aidrug.re.kr/web/, accessed on 18 July 2022). This tool quickly and accurately predicts ADMET properties of molecules using the 2D structure of the molecule (SMILES code) that is extremely helpful in making decisions that can determine the success of designed compounds. Hence, prediction results showed in Table 3 shows that designed inhibitors have promising ADMET properties. These inhibitors could be further evaluated using experimental studies and our design strategy can be used to develop potent and selective IRAK4 inhibitors.

## 3. Materials and Methods

### 3.1. Test Set/Training Set Selection for 3D-QSAR Analyses

A dataset of 38 IRAK4 inhibitors, with the diaminonicotinamide as a common scaffold, was selected for our study [2]. SYBYL-X 2.1 was used to draw and optimize the structures utilizing energy minimization with Tripos force field [26]. To create 3D-QSAR models, biological activities (IC_50_) were translated into pIC_50_ (-log IC_50_) values and used as dependent variables. The activity log span of pIC_50_ values of dataset compounds was more than 3 logarithmic units, that is within the necessity range [24,26]. The dataset was divided into a training set of 26 compounds for model generation and 12 compounds as test set for model validation based on the activity span of compounds. The chemical structures of the dataset compounds with their IC_50_ values are listed in Table S1 of supplementary material, in which the test set compounds are denoted by *.

### 3.2. Modeling of the Missing Residues

The crystal structure of IRAK4 with high resolution (PDB ID: 5W85) was obtained from the protein data bank for our study. Missing residues are present in the loop region from residue Ala216 to Thr223 and Glu337 to Gln341, which were modeled and refined using the modellerV9.14 [27]. Taking into consideration the energy, GA341 score [28], and DOPE score [29], the final modeled structure was selected. 

### 3.3. Preparation of the Protein and Molecular Docking

We used Autodock 4 to perform molecular docking of the most potent compound 33 of the series [30]. The co-crystal structure (PDB: 5W85) was utilized as a reference to dock the compound 33 inside the active site of the IRAK4 kinase. Before performing docking, the receptor structure was prepared by the addition of polar hydrogens, applying Kollman charges and assigning AD4 atom types. Subsequently, Autodock tools were used to prepare the ligand by keeping the number of rotatable bonds less than 6. The active site grid was produced using the x, y, and z coordinates of the co-crystallized ligand. The grid box was extended to 70 × 70 × 70 points, with a grid spacing of 0.375 Å. The docking was executed using the Lamarckian genetic algorithm (LGA) by setting the number of the genetic algorithm (GA) run to 100 [24]. The docked pose of compound 33 was selected based on its interactions with IRAK4 kinase and the lowest binding energy.

### 3.4. Molecular Dynamics Simulations

The MD simulation was performed using Gromacs-2018 [20]. The protein and ligand topology files were generated using Amber99SB force field [31] and general AMBER force field (GAFF) [32], respectively. The ligand force field parameters were developed using the ACPYPE program [33]. The system was neutralized by adding 13 sodium ions. A three-point water model (TIP3P) was used as the solvent. Energy minimization was performed by using the steepest descent method for 50,000 steps. Subsequently, the system was equilibrated first via a NVT ensemble for a 100 ps at 300 K using Berendsen thermostat [34] and then using NPT for 100 ps with the constant pressure of 1 atm. The bonds were constrained using the LINCS algorithm [35]. The particle mesh Ewald (PME) method [36] was utilized to handle the long-range coulombic interactions. A 50 ns production run was performed using NPT ensemble at 300 K with 1.0 atm pressure with a time step of 2 fs.

### 3.5. MM/PBSA Binding Free Energy Calculations

The g_mmpbsa package was used to perform molecular mechanics Poisson-Boltzmann surface area (MM/PBSA) free energy calculation [21]. The last 1 ns from the production run of 50 ns MD simulation was utilized for the calculation of binding free energy. The binding free energy contains three energetic terms, including potential energy in vacuum, polar-solvation energy, and nonpolar solvation energy. The molecular mechanics force field parameters were used to calculate both bonded (angle, bond, and dihedral) and non-bonded (electrostatic and van der Waal) interactions included in the potential energy in vacuum. Similarly, the Poisson-Boltzmann equation and solvent accessible surface area (SASA) model was used to calculate polar solvation energy and nonpolar solvation energy, respectively [37]. The assessment of binding free energy for the protein-ligand complex in a solvent was calculated based on the equation given below:

ΔG_binding_ = ΔG_complex_ − (ΔG_protein_ + ΔG_ligand_) (1)

where, ΔG_binding_ is the binding free energy and ΔG_complex_, ΔG_protein_, and ΔG_ligand_ represent the free energy of complex, protein, and ligand, respectively.

### 3.6. CoMFA and RF-CoMFA

3D-QSAR models were developed using Comparative Molecular Field Analysis (CoMFA) and Region-Focused CoMFA (RF-CoMFA) to correlate the biological activity with the 3D structure of the compounds using SYBYL-X 2.1. CoMFA utilizes the steric and electrostatic potential energies which are calculated using Lennard-Jones and Coulombic potentials respectively. The dataset compounds were aligned using a template molecule (50 ns MD pose of the most active compound 33) [24]. The selection of an appropriate partial charge scheme is important to develop reasonable 3D-QSAR models [24]. We have selected Pullman as partial charge scheme to generate CoMFA models. Default parameters were utilized to develop CoMFA and RF-CoMFA models. An sp^3^ hybridized carbon as probe atom with +1 charge and a grid spacing of 2.0 Å was used. Statistically reasonable CoMFA and RF-CoMFA models were developed using the Partial Least Squares (PLS) regression. In the PLS analysis, CoMFA descriptors and biological activity values (pIC_50_) were used as independent variables and dependent variables respectively. PLS analysis with Leave-one-out (LOO) crossvalidation was executed to evaluate the reliability of the generated models. PLS analysis was used to calculate the squared cross-validated correlation coefficient (*q*^2^) value, an optimal number of components (ONC) and the standard deviation of prediction (SEP). A column filtering value of 2.0 was used. Based on the obtained ONC, non-crossvalidation analysis was then performed to calculate the squared correlation coefficient (*r*^2^), F-test value (F) and standard error of estimate (SEE).

Similarly, RF-CoMFA model was generated using the PLS analysis obtained during CoMFA model development. RF-CoMFA is an iterative process that refines a built model by improving the weight for those lattice points which are most related to the model. This enhances the predictive capability of the PLS analysis used in RF-CoMFA.

#### Model Validation

The selected RF-CoMFA model was checked for predictive ability using different validation techniques such as bootstrapping, leave-five-out (LOF), an external test set validation, and *rm*^2^ metric calculations [25]. Bootstrapping for 100 runs was performed to validate the model’s predictability [38]. The models were also validated by the predictive correlation coefficient (*r*^2^*_pred_*).

### 3.7. Design of New IRAK4 Inhibitors and ADMET Calculation

We have derived a design strategy based on the structural information obtained from the contour map analysis of selected RF-CoMFA models. We designed new eight compounds and further calculated their in-silico ADMET (absorption, distribution, metabolism, excretion and toxicity), pharmacokinetic properties using Artificial Intelligence based In-silico ADME/Tox prediction online tool (https://www.aidrug.re.kr/web/ accessed on 18 July 2022). This tool quickly and accurately predicts ADMET properties of molecules using the 2D structure of the molecule (SMILES code) that is extremely helpful in making decisions that can determine the success of designed compounds.

## 4. Conclusions

IRAK4 is a one of the important serine/threonine kinases that play fundamental role in cell signaling, inflammation, apoptosis, and cellular differentiation, which makes it an ultimate drug target for several types of cancers and autoimmune diseases. In this study, we have employed various molecular modeling techniques, such as molecular docking, MD simulation, and MM/PBSA binding free energy calculation, in order to examine and to identify the essential active site residues accountable for IRAK4 inhibition. A comprehensive investigation showed that active site residues Val200, Ala211, Tyr262, Val26, Met265 and Leu318 were important for the IRAK4 inhibition. It was concluded from the MM/PBSA binding free energy calculation that residues Tyr262, Leu318 and Met265 were found to be involved more in the total binding energy. Moreover, RF-CoMFA resulted in reasonable statistical models in terms of *q*^2^ and *r*^2^ (*q*^2^ = 0.751, ONC = 4, *r*^2^ = 0.911). The model was found to be predictive and reliable. Our docking and MD results were compatible with the analysis of contour maps produced using a chosen RF-CoMFA model, thereby it elucidated the structural features requisite to design more potent IRAK4 inhibitors. We proposed a new design strategy based on the overall analysis and acquired structural features to modify the ligand and designed eight IRAK4 inhibitors. Our designed IRAK4 inhibitors exhibited predicted activity (pIC_50_) greater than the most potent compound of the diaminonicotinamide derivatives and their ADMET calculation showed promising results, which can be further evaluated using experimental studies for their specific contribution in the inhibition of IRAK4 as well as pharmacodynamics/pharmacokinetics properties. Our design scheme and predicted ADMET values could be useful for medicinal chemists or pharmaceutical companies to develop novel IRAK4 inhibitors.

## Figures and Tables

**Table 1:** Detailed statistical values of the selected RF-CoMFA model.

Parameter	RF-CoMFA	RF-CoMFA (Test Set 16)
*q* ^2^	0.527	0.751
ONC	6	4
SEP	0.694	0.395
*r* ^2^	0.905	0.911
SEE	0.258	0.236
F-value	73.716	53.689
*Q* ^2^	-	0.568
BS-*r*^2^	-	0.932
BS-SD	-	0.038
*r* ^2^ *_pred_*	-	0.808
LOF	-	0.751
*rm* ^2^	-	0.523
Δ *rm*^2^	-	0.120

*q*^2^: squared cross-validated correlation coefficient; ONC: optimal number of components; SEP: standard error of prediction; *r*^2^: squared correlation coefficient; SEE: standard error of estimation; F value: F-test value; LOF: leave-out-five; BS-*r*^2^: bootstrapping *r*^2^ mean; BS-SD: bootstrapping standard deviation; *r*^2^*_pred_*: predictive *r*^2^; *rm*^2^: average *rm*^2^ metric calculation; Δ *rm*^2^: standard error.

**Table 2:** The structures and the predicted pIC_50_ values of the designed IRAK4 antagonists.

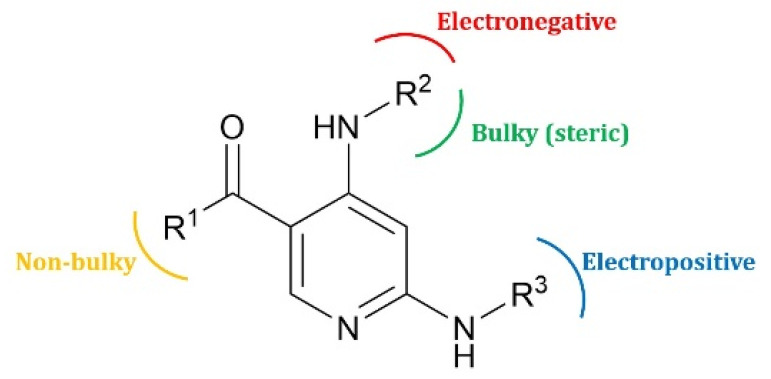				
**Compound**	**R^1^**	**R^2^**	**R^3^**	**Predicted pIC_50_**
D01	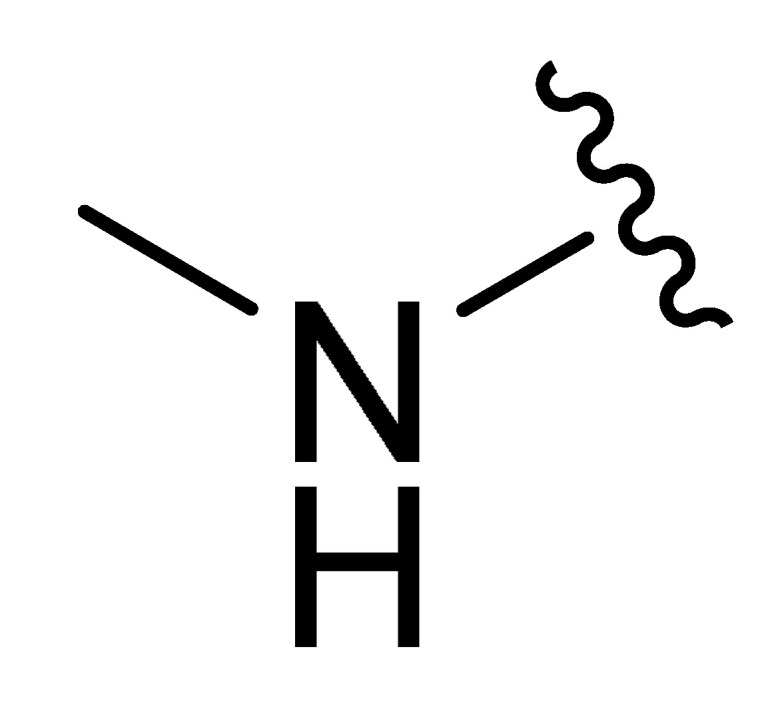	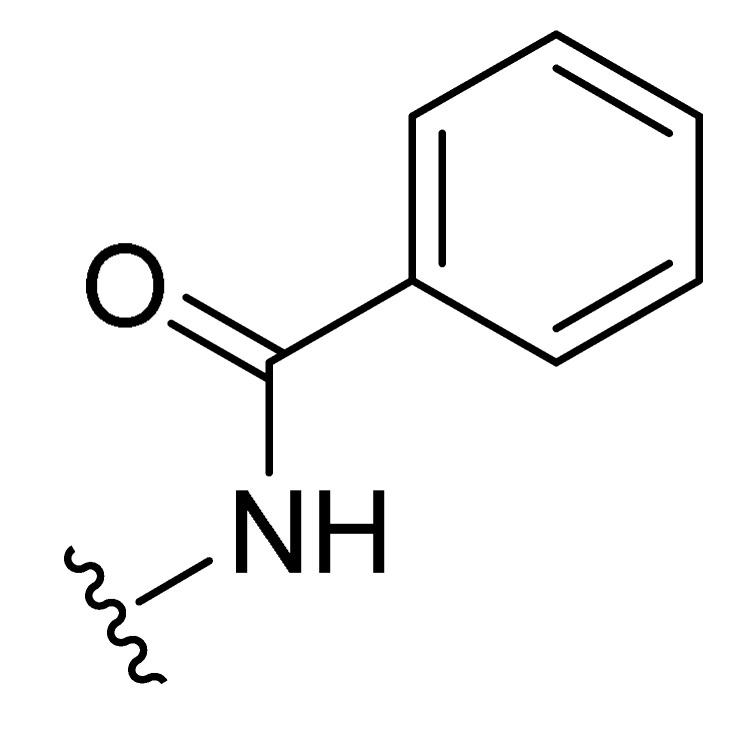	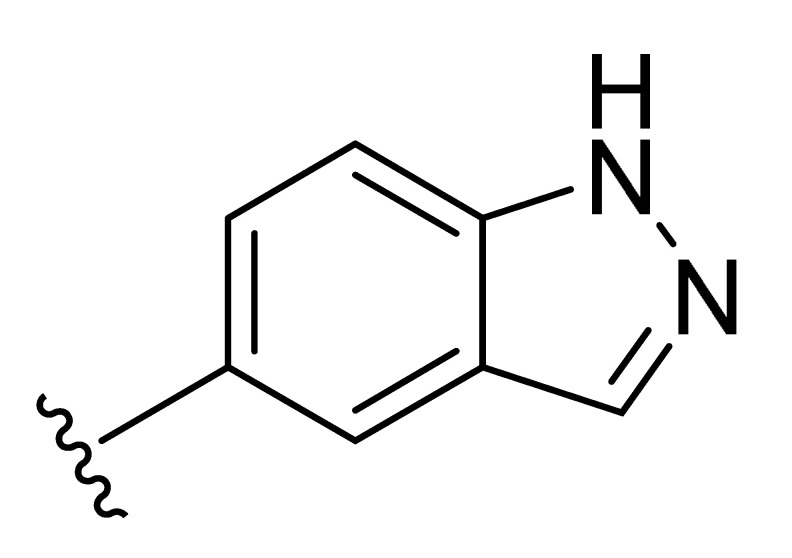	8.20
D02		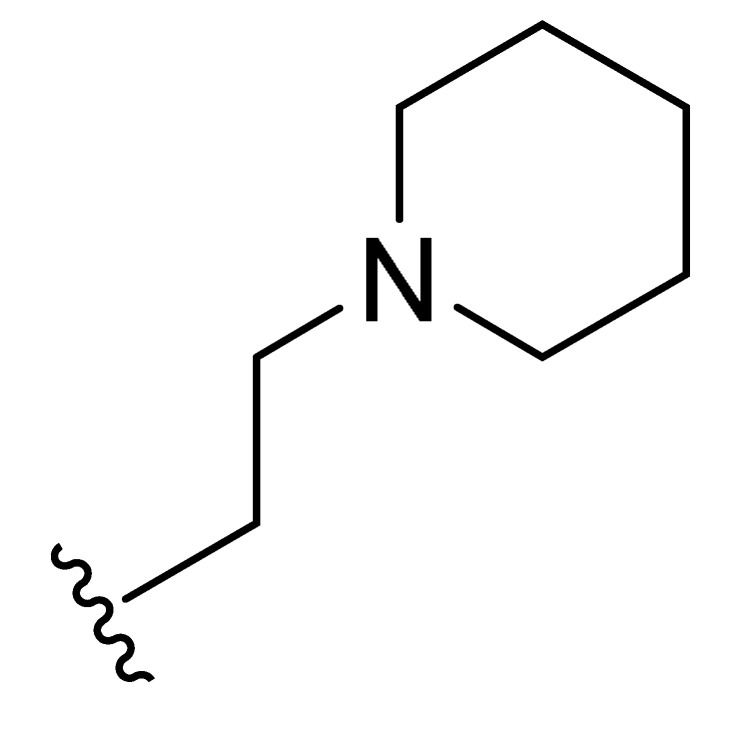		8.236
D03		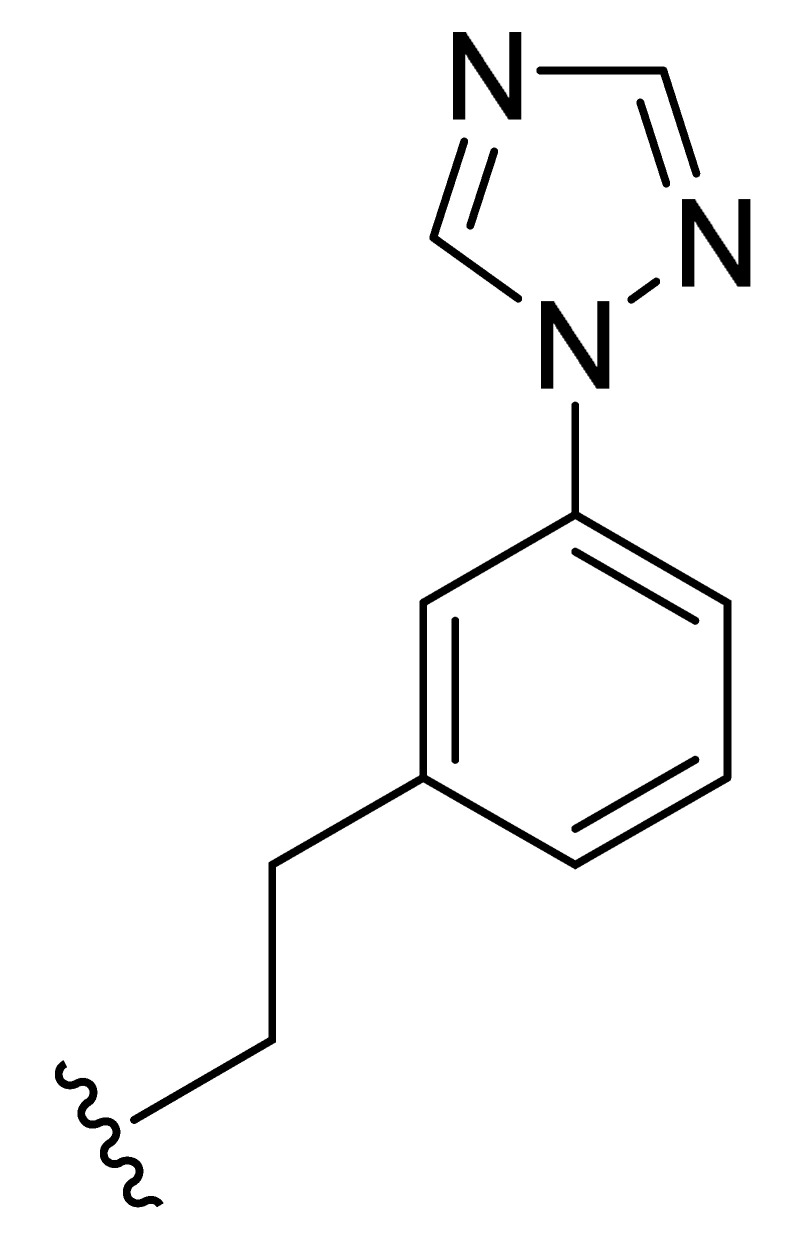		8.612
D04		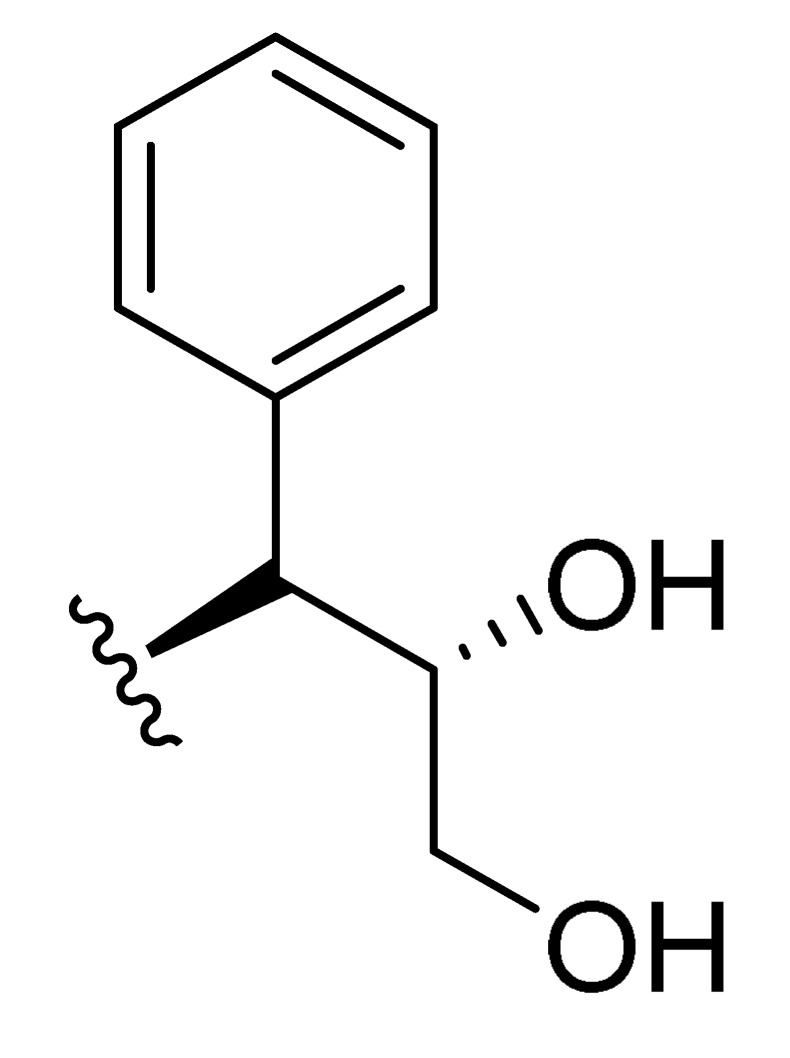	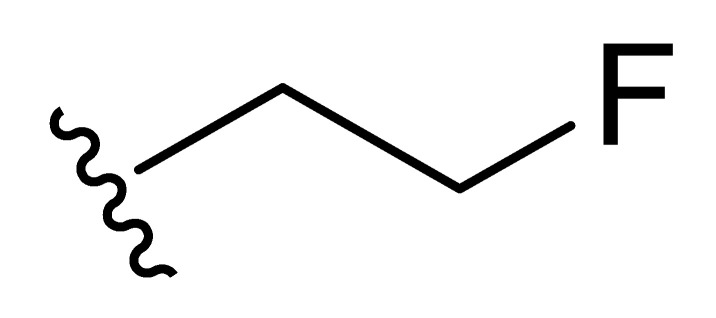	8.50
D05			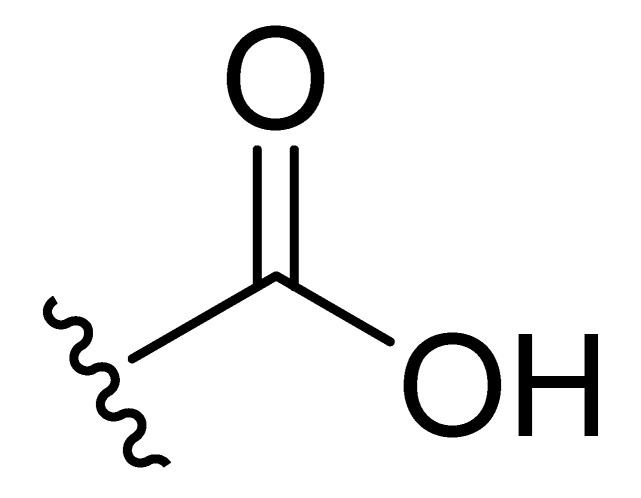	8.289
D06			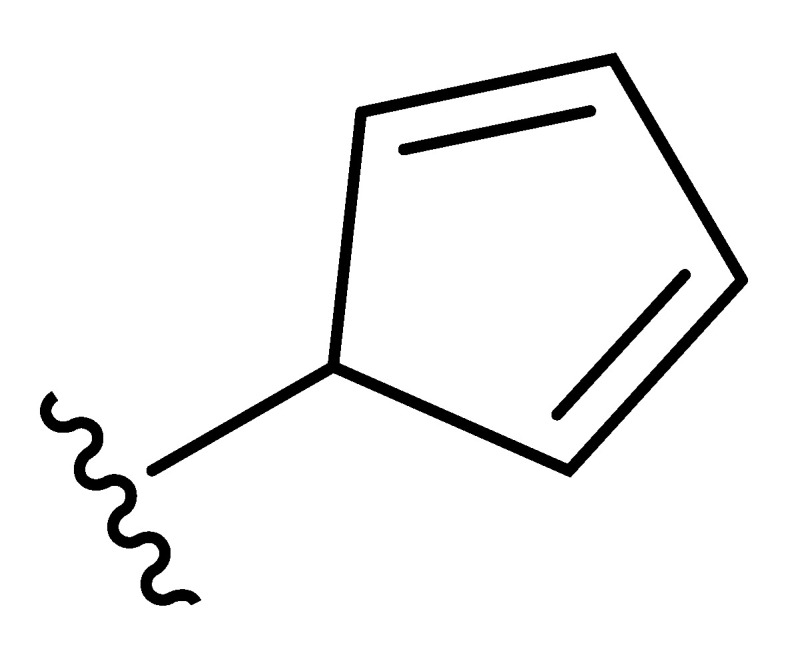	8.02
D07			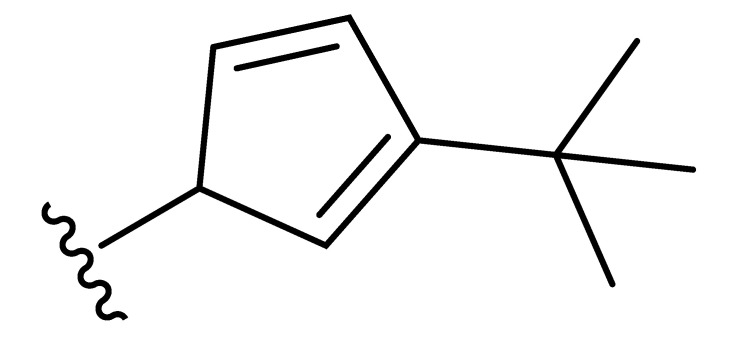	8.32
D08			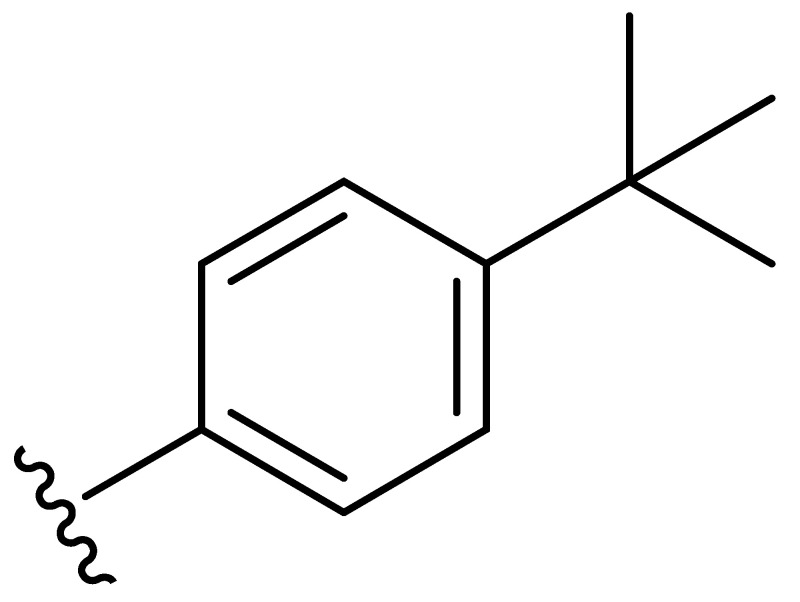	8.30

**Table 3:** In silico ADMET prediction and synthetic accessibility values of designed IRAK4 inhibitors.

Compound	Properties		Absorption (in %)	Distribution (in %)		Metabolism (in %)					Elimination (Liver Microsomal Stability) (in %)			Toxicity (in %)
	TPSA (in %)	AlogP	Passive Absorption (Permeability)	Blood-Brain Barrier Penetration	P-gp Substrates	CYP1A2 Inhibition	CYP2D6 Inhibition	CYP2C9 Inhibition	CYP2C19 Inhibition	CYP3A4 Inhibition	Human	Mouse	Rat	hERG Inhibition
D1	111.80	3.36	52	67	39	55	34.50	57	49	44	53.38	77	66	48.43
D2	97.97	2.96	55	65.50	43	53	39	57.20	57	47	51.48	76	61	54.33
D3	125.44	3.30	46	57	42	56	32	49.60	44.67	48	54.28	71	59	51.28
D4	106.51	1.33	55	65	28	53.50	22.33	56.10	47.08	41	54.46	76	63	43.17
D5	143.81	1.04	44	68	45	45.50	19.50	56.90	50.33	43	53.19	79	69	41.17
D6	106.51	1.85	58	61	37	50.50	23.33	55.20	49.89	44	50.63	78	52	43.63
D7	106.51	3.27	55	59	41	45	27.33	54.80	54.33	49	54.21	73	56	46.30
D8	106.51	3.99	50	56	38	46	29.17	55.40	48.67	43	48	78	63	50.30

## Data Availability

Data is contained within the article and supplementary material.

## Supplementary Materials

The following supporting information can be downloaded at: https://www.mdpi.com/article/10.3390/molecules27196307/s1, Table S1: The chemical structures of the selected IRAK4 inhibitors with their IC_50_ values; Table S2: The predicted pIC_50_ and residual values for RF-CoMFA model.

## References

[1] Kargbo R.B. (2019). PROTAC Degradation of IRAK4 for the Treatment of Cancer. ACS Med. Chem. Lett..

[2] Bhide R.S., Keon A., Weigelt C., Sack J.S., Schmidt R.J., Lin S., Xiao H.-Y., Spergel S.H., Kempson J., Pitts W.J. (2017). Discovery and structure-based design of 4,6-diaminonicotinamides as potent and selective IRAK4 inhibitors. Bioorgan. Med. Chem. Lett..

[3] Chaudhary D., Robinson S., Romero D.L. (2015). Recent advances in the discovery of small molecule inhibitors of interleukin-1 receptor-associated kinase 4 (IRAK4) as a therapeutic target for inflammation and oncology disorders: Miniperspective. J. Med. Chem..

[4] Rhyasen G.W., Starczynowski D.T. (2015). IRAK signalling in cancer. Br. J. Cancer.

[5] Warner N., Núñez G. (2013). MyD88: A Critical Adaptor Protein in Innate Immunity Signal Transduction. J. Immunol..

[6] Buckley G.M., Gowers L., Higueruelo A.P., Jenkins K., Mack S.R., Morgan T., Parry D.M., Pitt W.R., Rausch O., Richard M.D. (2008). IRAK-4 inhibitors. Part 1: A series of amides. Bioorgan. Med. Chem. Lett..

[7] Genung N., Guckian K. (2017). Small Molecule Inhibition of Interleukin-1 Receptor-Associated Kinase 4 (IRAK4). Prog. Med. Chem..

[8] Dunne A., Carpenter S., Brikos C., Gray P., Strelow A., Wesche H., Morrice N., O’Neill L. (2010). IRAK1 and IRAK4 Promote Phosphorylation, Ubiquitination, and Degradation of MyD88 Adaptor-like (Mal). J. Biol. Chem..

[9] Flannery S., Bowie A.G. (2010). The interleukin-1 receptor-associated kinases: Critical regulators of innate immune signalling. Biochem. Pharmacol..

[10] Medvedev A.E., Lentschat A., Kuhns D.B., Blanco J., Salkowski C., Zhang S., Arditi M., Gallin J.I., Vogel S.N. (2003). Distinct Mutations in IRAK-4 Confer Hyporesponsiveness to Lipopolysaccharide and Interleukin-1 in a Patient with Recurrent Bacterial Infections. J. Exp. Med..

[11] Khanfar M.A., Alqtaishat S. (2019). Discovery of potent IRAK-4 inhibitors as potential anti-inflammatory and anticancer agents using structure-based exploration of IRAK-4 pharmacophoric space coupled with QSAR analyses. Comput. Biol. Chem..

[12] Srivastava R., Geng D., Liu Y., Zheng L., Li Z., Joseph M.A., McKenna C., Bansal N., Ochoa A., Davila E. (2012). Augmentation of Therapeutic Responses in Melanoma by Inhibition of IRAK-1,-4IRAK-4 Signaling and Melanoma Progression. Cancer Res..

[13] Khurana N., Dodhiawala P.B., Bulle A., Lim K.-H. (2020). Deciphering the role of innate immune NF-ĸB pathway in pancreatic cancer. Cancers.

[14] Rahib L., Smith B.D., Aizenberg R., Rosenzweig A.B., Fleshman J.M., Matrisian L.M. (2014). Projecting cancer incidence and deaths to 2030: The unexpected burden of thyroid, liver, and pancreas cancers in the United States. Cancer Res..

[15] Li Q., Chen Y., Zhang D., Grossman J., Li L., Khurana N., Jiang H., Grierson P.M., Herndon J., DeNardo D.G. (2019). IRAK4 mediates colitis-induced tumorigenesis and chemoresistance in colorectal cancer. JCI Insight.

[16] Poso A., von Wright A., Gynther J. (1995). An empirical and theoretical study on mechanisms of mutagenic activity of hydrazine compounds. Mutat. Res. Mol. Mech. Mutagen..

[17] Seganish W.M. (2016). Inhibitors of interleukin-1 receptor-associated kinase 4 (IRAK4): A patent review (2012–2015). Expert Opin. Ther. Patents.

[18] McElroy W.T. (2019). Interleukin-1 receptor-associated kinase 4 (IRAK4) inhibitors: An updated patent review (2016–2018). Expert Opin. Ther. Pat..

[19] Eisenberg D., Schwarz E., Komaromy M., Wall R. (1984). Analysis of membrane and surface protein sequences with the hydrophobic moment plot. J. Mol. Biol..

[20] Lemkul J. (2018). From Proteins to Perturbed Hamiltonians: A Suite of Tutorials for the GROMACS-2018 Molecular Simulation Package [Article v1.0]. Living J. Comput. Mol. Sci..

[21] Kumari R., Kumar R., Consortium O.S.D.D., Lynn A.J. (2014). modeling. g_mmpbsa A GROMACS tool for high-throughput MM-PBSA calculations. J. Chem. Inf. Modeling.

[22] Cramer R.D., Patterson D.E., Bunce J.D. (1988). Comparative molecular field analysis (CoMFA). 1. Effect of shape on binding of steroids to carrier proteins. J. Am. Chem. Soc..

[23] Golbraikh A., Tropsha A. (2000). Predictive QSAR modeling based on diversity sampling of experimental datasets for the training and test set selection. Mol. Divers..

[24] Gadhe C.G., Kothandan G., Cho S.J. (2012). Large variation in electrostatic contours upon addition of steric parameters and the effect of charge calculation schemes in CoMFA on mutagenicity of MX analogues. Mol. Simul..

[25] Roy K., Chakraborty P., Mitra I., Ojha P.K., Kar S., Das R.N. (2013). Some case studies on application of “rm2” metrics for judging quality of quantitative structure–activity relationship predictions: Emphasis on scaling of response data. J. Comput. Chem..

[26] Thibaut U., Folkers G., Klebe G., Kubinyi H., Merz A., Rognan D. (1994). Recommendations for CoMFA Studies and 3D QSAR Publications. Quant. Struct. Relatsh..

[27] Webb B., Sali A. (2021). Protein structure modeling with MODELLER. Structural Genomics.

[28] Melo F., Sánchez R., Sali A. (2002). Statistical potentials for fold assessment. Protein Sci..

[29] Shen M.-Y., Sali A. (2006). Statistical potential for assessment and prediction of protein structures. Protein Sci..

[30] Huey R., Morris G., Olson A.J., Goodsell D.S. (2007). A semiempirical free energy force field with charge-based desolvation. J. Comput. Chem..

[31] Hornak V., Abel R., Okur A., Strockbine B., Roitberg A., Simmerling C. (2006). Comparison of multiple Amber force fields and development of improved protein backbone parameters. Proteins: Struct. Funct. Bioinform..

[32] Wang J., Wolf R.M., Caldwell J.W., Kollman P.A., Case D.A. (2004). Development and testing of a general amber force field. J. Comput. Chem..

[33] Sousa da Silva A.W., Vranken W.F. (2012). ACPYPE-Antechamber python parser interface. BMC Res. Notes.

[34] Berendsen H.J.C., Postma J.P.M., Van Gunsteren W.F., DiNola A., Haak J.R. (1984). Molecular dynamics with coupling to an external bath. J. Chem. Phys..

[35] Hess B. (2008). P-LINCS: A Parallel Linear Constraint Solver for Molecular Simulation. J. Chem. Theory Comput..

[36] Essmann U., Perera L., Berkowitz M.L., Darden T., Lee H., Pedersen L.G. (1995). A smooth particle mesh Ewald method. J. Chem. Phys..

[37] Gohlke H., Kiel C., Case D.A. (2003). Insights into Protein–Protein Binding by Binding Free Energy Calculation and Free Energy Decomposition for the Ras–Raf and Ras–RalGDS Complexes. J. Mol. Biol..

[38] Chirico N., Gramatica P. (2012). Real External Predictivity of QSAR Models. Part 2. New Intercomparable Thresholds for Different Validation Criteria and the Need for Scatter Plot Inspection. J. Chem. Inf. Model..

