# Supplementary material for: Design of Novel IRAK4 Inhibitors Using Molecular Docking, Dynamics Simulation and 3D-QSAR Studies
Source: Molecules. 2022 Sep 24;27(19):6307. doi: 10.3390/molecules27196307 (PMC9570937; doi:10.3390/molecules27196307)
Supplement: Supplementary file 1 [file molecules-27-06307-s001.zip › molecules-1896744-supplementary.pdf]

Supplementary Material

Table S1. The chemical structures and activity values (IC_{50}) of the selected 4,6- diaminonicotinamide IRAK4 antagonists.

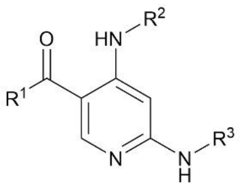

Compound	R^1	R^2	R^3	IC_{50} (nM)
1	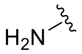	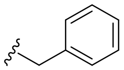	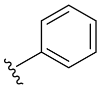	1800
2*		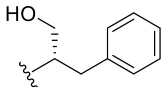		730
3		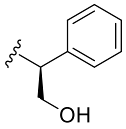		840
4		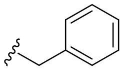	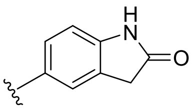	360
5*			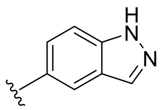	390

6			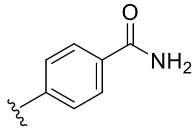	470
7*			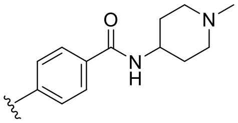	10000
8*		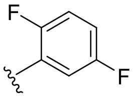	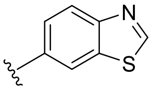	180
9*		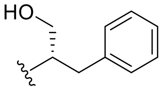		19
10	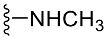			10
11	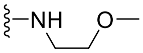			64
12*	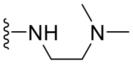			50
13	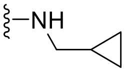			70
14	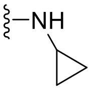			50
15*	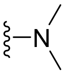			14600
16*	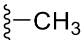	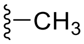		730
17*		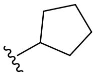		13
18*		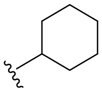		20
19		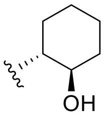		47
20		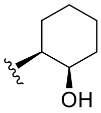		130
21*		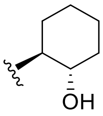		82
22		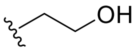		170
23		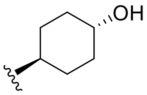		40

24		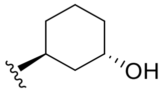		25
25		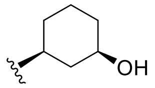		11
26		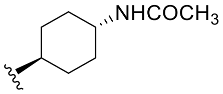		21
27*		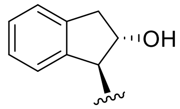		2000
28		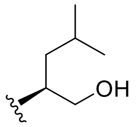		50
29				5
30	H			20
31			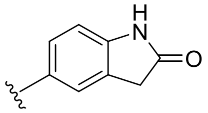	5
32			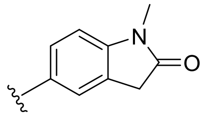	10
33			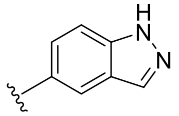	4
34	$-NHCH_3$	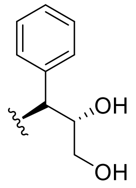	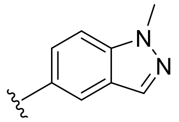	13
35			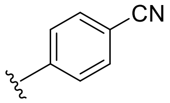	5
36			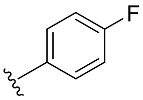	10
37			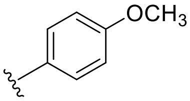	10
38			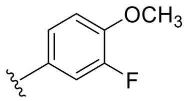	13

*Test set compounds

Table S2: The predicted pIC_{50} and residual values for the RF–CoMFA model.

Compound	Actual pIC_{50}	RF–CoMFA	
		Predicted pIC_{50}	Residual
01	5.745	6.043	-0.298
02	6.137	6.919	-0.782
03	6.076	6.001	0.075
04	6.444	6.37	0.074
05	6.409	6.231	0.178
06	6.328	6.192	0.136
07	5	5.931	-0.931
08	6.745	6.192	0.553
09	7.721	7.081	0.64
10	8	7.481	0.519
11	7.194	7.268	-0.074
12	7.301	7.393	-0.092
13	7.155	7.247	-0.092
14	7.301	7.406	-0.105
15	4.836	5.250	-0.414
16	6.137	6.588	-0.451
17	7.886	6.906	0.98
18	7.699	7.345	0.354
19	7.328	7.317	0.011
20	6.886	7.477	-0.591
21	7.086	7.526	-0.44
22	6.77	6.62	0.15
23	7.398	7.529	-0.131
24	7.602	7.503	0.099
25	7.959	7.573	0.386
26	7.678	7.673	0.005
27	5.699	6.198	-0.499
28	7.301	7.473	-0.172
29	8.301	8.118	0.183
30	7.699	7.818	-0.119
31	8.301	8.406	-0.105
32	8	7.946	0.054
33	8.398	8.213	0.185
34	7.886	7.796	0.09
35	8.301	8.253	0.048
36	8	7.973	0.027
37	8	8.115	-0.115
38	7.886	8.128	-0.242
